# Pediatric bone and soft-tissue sarcomas

**DOI:** 10.1186/1470-7330-15-S1-O36

**Published:** 2015-10-02

**Authors:** Beth McCarville

**Affiliations:** 1 Department of Diagnostic Imaging, St. Jude Children's Research Hospital, 262 Danny Thomas Place, MS 220, Memphis, TN 38105, USA

## 

In this session I will review the clinical and imaging features of the most common pediatric bone and soft tissue sarcomas and several benign lesions that may mimic them. These malignancies often have unique features that can help narrow the differential diagnosis. The material will be presented in an informal manner and audience participation is encouraged. A review of the appropriate diagnostic work-up will also be presented, including the role of various imaging modalities to detect local-regional and distant metastatic disease. At the end of this session participants should be able to identify clinical features (age, race, etc.) and imaging features that are suggestive of the correct diagnosis.
